# Alteration in the Lipid Profile and the Desaturases Activity in Patients With Severe Pneumonia by SARS-CoV-2

**DOI:** 10.3389/fphys.2021.667024

**Published:** 2021-05-11

**Authors:** Israel Pérez-Torres, Verónica Guarner-Lans, Elizabeth Soria-Castro, Linaloe Manzano-Pech, Adrián Palacios-Chavarría, Rafael Ricardo Valdez-Vázquez, Jose Guillermo Domínguez-Cherit, Hector Herrera-Bello, Humberto Castillejos-Suastegui, Lidia Moreno-Castañeda, Gabriela Alanís-Estrada, Fabián Hernández, Omar González-Marcos, Ricardo Márquez-Velasco, María Elena Soto

**Affiliations:** ^1^Departament of Cardiovascular Biomedicine, Instituto Nacional de Cardiología Ignacio Chávez, Mexico City, Mexico; ^2^Departament of Physiology, Instituto Nacional de Cardiología Ignacio Chávez, Mexico City, Mexico; ^3^Critical Care Unit of the Temporal COVID-19 Unit, Citibanamex Center, Mexico City, Mexico; ^4^American British Cowdray Medical Center, Mexico City, Mexico; ^5^Instituto Nacional de Ciencias Médicas y Nutrición Salvador Zubirán, Mexico City, Mexico; ^6^Tecnológico de Monterrey EMCS, Mexico City, Mexico; ^7^Departament of Immunology, Instituto Nacional de Cardiología Ignacio Chávez, Mexico City, Mexico

**Keywords:** COVID-19, fatty acids, SARS-CoV-2, NEFAs, phospholipids, pneumonia

## Abstract

The kidnapping of the lipid metabolism of the host’s cells by severe acute respiratory syndrome (SARS-CoV-2) allows the virus to transform the cells into optimal machines for its assembly and replication. Here we evaluated changes in the fatty acid (FA) profile and the participation of the activity of the desaturases, in plasma of patients with severe pneumonia by SARS-CoV-2. We found that SARS-CoV-2 alters the FA metabolism in the cells of the host. Changes are characterized by variations in the desaturases that lead to a decrease in total fatty acid (TFA), phospholipids (PL) and non-esterified fatty acids (NEFAs). These alterations include a decrease in palmitic and stearic acids (*p* ≤ 0.009) which could be used for the formation of the viral membranes and for the reparation of the host’s own membrane. There is also an increase in oleic acid (OA; *p* = 0.001) which could modulate the inflammatory process, the cytokine release, apoptosis, necrosis, oxidative stress (OS). An increase in linoleic acid (LA) in TFA (*p* = 0.03) and a decreased in PL (*p* = 0.001) was also present. They result from damage of the internal mitochondrial membrane. The arachidonic acid (AA) percentage was elevated (*p* = 0.02) in the TFA and this can be participated in the inflammatory process. EPA was decreased (*p* = 0.001) and this may decrease of pro-resolving mediators with increase in the inflammatory process. The total of NEFAs (*p* = 0.03), PL (*p* = 0.001), cholesterol, HDL and LDL were decreased, and triglycerides were increased in plasma of the COVID-19 patients. Therefore, SARS-CoV-2 alters the FA metabolism, the changes are characterized by alterations in the desaturases that lead to variations in the TFA, PL, and NEFAs profiles. These changes may favor the replication of the virus but, at the same time, they are part of the defense system provided by the host cell metabolism in its eagerness to repair damage caused by the virus to cell membranes.

## Introduction

Out of the 36 coronaviruses that belong to the *Coronaviridae* family, the β-coronavirus type 2 it is one that causes the severe acute respiratory syndrome (SARS−CoV−2) infection in humans. This infection is associated with acute lung injury (ALI), that may rapidly progress to acute respiratory distress syndrome (ARDS), leading to COVID−19. The clinical presentations of COVID−19 range from asymptomatic cases to individuals with severe pneumonia associated with ARDS and cardiogenic shock. The severe forms are more often present in patients with chronic comorbidities including the pathologies that comprise the metabolic syndrome and in the elderly population (Soto et al., 2020).

Lipid interactions are especially important during the entrance of the viruses to the host cell and in the infection process. Fatty acids (FAs) constitute the main components of the cellular plasma membrane, the organelles, and the vesicles that store energy and they are the building blocks of lipid metabolism. They are also the first line of defense when encountering pathogens. Without FA, life could not be possible (Van Meer et al., 2008). The kidnapping of the lipid metabolism by viruses allows the remodeling of the host cells, transforming them into machines for the benefit of the virus (Heaton and Randall, 2011). Viral membranes synthesized by the altered lipid metabolism are later decorated with nucleocapsid and spike proteins, as the virus is assembled in the inner part of the cell membrane before it buds to infect the neighboring cells (Freed, 2002). The budding process allows the viral particles to leave the cell without breaking the host’s plasma membrane. The infection process of the virus depends on its membrane to facilitate its entrance to the host cell through endocytosis. The viral membrane also helps in bypassing the immune response by avoiding the recognition of the viral proteins, prolonging survival, and enhancing the spread of the virus (Thomas et al., 2020a). As an example, the Dengue virus kidnaps the host FA synthase sites to use them for its own replication and to further synthesize FA for survival (Shaath and Alajez, 2020).

Although there are many studies on the epidemiological and clinical characteristics in COVID-19 patients, relatively few studies have focused on the assembly and replication mechanisms of the virus despite the enormous consequences they it may have for the host. The study of the proteome, lipidome, and metabolome in human biofluids from patients infect by SARS-Cov-2, provides insight into the viral cycle, the state of the disease, the molecules that play essential roles in COVID-19 and possible therapeutic targets. Although alterations caused by SARS-CoV-2 on the metabolome and lipidome in human plasma have been analyzed and the results indirectly provide information on the physiological mechanisms of the viral replication, there are still important gaps to be understood, Shaath and Alajez, 2020). These studies have not addressed the role of desaturases in the infection process. Therefore, the purpose of this study was evaluated changes in the FA profile and the participation of the desaturases in plasma of patients with severe pneumonia by SARS-CoV-2.

## Materials and Methods

The study population consisted of patients over 18 years of age who were admitted to the ICU of the CITIBANAMEX Center and who may have developed or not septic shock, secondary to moderate or severe pneumonia due to COVID-19. The diagnostic criteria for septic shock were based on the Sepsis-3 consensus (Olson and Andrew, 2020). Patients considered to have septic shock had to have an acute increase of at least two points in the SOFA score (Lambden et al., 2019), lactate levels greater than 2 mmol/L and they had to be dependent on a vasopressor for at least 2 h before the time of enrollment. Exclusion from this study occurred when patients were younger than 18 years, when they were not able to grant an informed consent, or when they refused to be included. Patients were also excluded if pregnant or breastfeeding or if they were under chronic use (last 6 months) or recent use of steroids, statins, or antioxidants.

The hospitalized patients included, were considered to have severe symptoms considering their ventilatory status. Patients with the severe condition required invasive mechanical intubation according to the criteria of Berlin for ARDS. The Berlin definition proposes 3 categories of ARDS based on the severity of hypoxemia: mild (200 mm Hg < Pao2/Fio2 ≤ 300 mm Hg), moderate (100 mm Hg < Pao2/Fio2 ≤ 200 mm Hg), and severe (Pao2/Fio2 ≤ 100 mm Hg), along with explicit criteria related to timing of the syndrome’s onset, origin of edema, and the chest radiographic findings. The ARDS definition task force was considered (Ranieri et al., 2012).

Twenty-two healthy subjects (HS) were matched by age and gender. HS were negative for SARS-CoV-2. The collection of peripheral blood samples was done by venopunction. In these subjects, there was no suspicion of inflammatory disease or presence of degenerative disorders such as thyroid and autoimmune diseases, diabetes mellitus, dyslipidemia, and arterial hypertension. The intake of some medications that could interfere with the results of the study as antioxidants drugs and NSAIDs was considered, and the drugs were suspended 48 h before the obtainment of the sample. Control subjects reported not having any disease. Nevertheless, after results were obtained, we found that two patients had moderately high levels of lipids, but they were not receiving treatment.

Ethical approval to perform the study was obtained from the local ethics committee on August 19, 2020 (Control-9867/2020, register REG. CONBIOETICA-09-CEI-011-20160627). A written informed consent for enrollment or consent to use data from the patients was obtained directly from them or their legal surrogate. The protocol was registered (TRIAL REGISTRATION: ClinicalTrials.gov Identifier: NCT 04570254). During the pre-treatment and post-treatment evaluation of the study (Chavarría et al., 2021), we observed that the lipid levels were below the normal range since the admission of the patients. It was therefore considered that, in addition to the main objective, the evaluation if this decrease needed to be explored as part of the mechanism of damage by SARS-CoV-2.

### Collection of Samples to Verify Infection by SARS-CoV-2

Paired saliva and nasopharyngeal swab samples were collected from 42 patients who were suspected to be infected by SARS-CoV-2. Samples were classified as positive for SARS-CoV-2 when both the N1 and N2 primer-probe sets were detected. The presence of the SARS-CoV-2 virus was evaluated using specific probes for the detection of the virus in conjunction with the real-time reverse transcriptase polymerase chain reaction technique (qRT-PCR). To evaluate organ dysfunction, the SOFA score (neurologic, respiratory, hemodynamic, hepatic, and hematologic) was calculated at admission and during the days of treatment (Lambden et al., 2019).

### Blood Sample Obtainment and Storage

Blood samples were obtained from each patient that entered the hospital. The blood samples were centrifuged for 20 min at 936 g and 4°C. The plasma of the samples was placed in 3 or 4 aliquots and stored at −30°C. Laboratory tests were made in patients with COVID-19 to determine acute-phase reactants, hemoglobin, leukocytes, lymphocytes, platelets, creatinine, urea nitrogen, glucose, C-reactive protein (CRP), albumin, D-dimer, ferritin, fibrinogen, procalcitonin (PCT), interleukin-6 (IL-6) and oxygen saturation. Data from the patient’s medical history including demographic, prior illnesses to infection by SARS-CoV-2, test result for COVID-19, whether mechanical ventilation was used, and type of treatment given were used for the analysis of the results. Additionally, total fatty acid (TFA), phospholipids (PL), FA of the phospholipids (FAPL) and non-esterified FA (NEFAs) were determined in plasma.

### Total Fatty Acid Determination

For the extraction and derivatization of the TFA, 100 μl of plasma were used in the presence of 50 μg of non-adecanoic acid (C17:0) as internal standard and 2 ml of chloroform-methanol (2:1, vol/vol) with 0.002% BHT were added, as described by the Folch method (Folch et al., 1957). TFA were trans esterified to their FA methyl esters by heating them at 90°C for 2 h with 2 ml of methanol plus 0.002% BHT, 40 μl of H_2_SO_4_ and 100 μl toluene. The TFA methyl esters were separated and identified by gas chromatography-FID in a Carlo Erba Fratovap 2300 chromatograph equipped with a capillary column packed with the stationary phase: HP-FFAP (Description: 30 m length × 0.320 mm diameter × 0.25 μm film) and fitted with a flame ionization detector at 210°C, with helium as the carrier gas at a flow rate of 1.2 ml/min and. The areas under of the peaks were calculated by a Shimadzu C-R6A Chromatopac integrator coupled to the gas chromatograph. The identification of each FA methyl ester was made by comparing their retention time to that of their corresponding standard.

### Fatty Acid of the Phospholipids Determination

For FAPL extraction, 200 μl of plasma were used in the presence of 50 μg of L-α-phosphatidylcholine-di-heptadecanoyl acid as internal standard and 1 ml of acetone. The mixture was shaken vigorously in a vortex for 30 s and centrifuged at 1145g, at room temperature for 4 min. The supernatant was removed, and the button was suspended with chloroform-methanol (2:1, vol/vol) with 0.002% BHT, as described by the Folch method (Folch et al., 1957). FAPL were trans esterified to their FAPL methyl esters and separated as described above.

### Total Phospholipids

The total phospholipids (TPL) determination in plasma was made by a colorimetric assay through an enzymatic method utilizing N-ethyl-N-(2, hydroxy-3-sulfopropyl)-3,5-domethoxyaniline, according to the manufacturer’s recommendations (Fujifilm; Phospholipids C, Code No. 997-01801, FUJIFLIM Wako Diagnostic U.S.A. Corporation). The absorbance was measured at 600 nm.

### Extraction and Derivatization NEFAs

A total of 100 μl of plasma was used in the presence of 50 μg of non-adecanoic acid as internal standard, 2 mL of chloroform methanol (2:1, vol/vol) with 0.002% BHT, as was described by Folch method (Folch et al., 1957). The obtained lipid residue was dissolved at room temperature for 15 min in 1 mL of methanol containing 100 μL of 2,2-dimethoxypropane and 10 μl of concentrated H_2_SO_4_ to esterify NEFAs to their corresponding methyl esters as described by Tserng et al. (1981). These reaction conditions are necessary to avoid the esterification of FA from PL, cholesterol esters, and triglycerides. The concentration and composition of NEFAs methyl esters was identified as described above.

### Total Non-esterified Fatty Acid

The total non-esterified fatty acid (TNEFAs) determination in plasma was made by a colorimetric kit according to the manufacturer’s recommendations (Sigma-Aldrich, Free Fatty Acid Quantitation Kit cat # MAK044 United States) The absorbance was measured at 492–630 nm.

### Statistical Analysis

Continuous variables were expressed as the mean ± standard deviation or median with minimum and maximum, depending on the distribution. Categorical variables were expressed as frequencies and percentages. The normality of the variables was evaluated using the Shapiro-Wilk or Shapiro-France test, depending on the size of the sample. A graphical analysis of the distribution of the variables was also performed with histograms and/or stem and leaf graphs. We used non-parametric tests (Mann–Whitney *U* test, Kruskal–Wallis *t*, depending on the particular case) to contrast variables without Gaussian distribution. The Pearson correlation was used for the estimation of biomarkers. Statistical analyses were performed with SPSS version 26. The Sigma Plot 14 program (Systat Software Inc. 2107, San Jose, CA95131 EE.UU. North First Street, Suite 360) was used to generate the analysis and graphs of the TFA, TPL and NEFAs. Statistical significance was determined by the Mann-Whitney rank sum test followed by the normality test (Shapiro-Wilk). Differences were considered statistically significant when *p* ≤ 0.05.

## Results

### Demographic Characteristics

A total of 42 covid-19 patients were examined, of which 31 (74%) were men and 11 (26%) were women. Patients had an age range of 62 ± 13 years. In all patients, infection by SARS-CoV-2 was diagnosed through a CRP-test. The average body mass index was 29 ± 4 kg/m^2^ with normal weight. Comorbid conditions prior to SARS-CoV-2 infection were, dyslipidemia 16 (38%), systemic arterial hypertension 19 (45%) diabetes mellitus 18 (43%), chronic obstructive lung disease 3 (7%), chronic kidney disease 4 (10%), coronary heart disease 1 (2%). Other variables include temperature 36.6 ± 0.46°C, arterial blood oxygen pressure (PaO_2_) 79.4 ± 38.4, partial pressure of carbon dioxide (PCO_2_) 33.1 ± 7.8, Kirby’s index which is PaCO_2_/inspired fraction of oxygen (FiO_2_) 123 ± 58.2, oxygen saturation (SpO_2_)/FiO_2_ 123.6 ± 52, FC 77 ± 20, mean arterial pressure 79 ± 11 mmHg, glucose 160.1 ± 77 mg/dL, urea 56.8 ± 53, ureic nitrogen 25.5 ± 22.8, total cholesterol (TC) 138.3 ± 38.4 mg/dL, triglycerides 155.2 ± 81.7 mg/dL, high density lipoprotein 33.1 ± 8.3 mg/dL, low density lipoprotein 75.7 ± 30.1 mg/dL, lactic dehydrogenase 314.7 ± 100.2 UI/L, total bilirubin 0.66 ± 0.20, direct bilirubin 0.22 ± 0.09, leukocytes 11.4 ± 4.8 10^3^/μL, lymphocytes 0.67 ± 0.40 10^3^/μL, platelets 243.07 ± 87 10^3^/μL, ferritin 836.80 ± 757 ng/mL, CRP median 151 with 32 range min and 384 range max, index neutrophil/lymphocyte median 13 with 1 range min and 106 range max, interleukin 6 median 117.04 with 7.8 range min and 638.5 range max pg/ml, D-dimer median 900 with 210 range min and 34920 range max μg/mL, Table 1.

**TABLE 1:** Demographic characteristic of COVID-19 patients at admission.

**Reference value units**	**Number and percentage**
Woman	11 (26)
Men	31 (74)
DM	18 (43)
SAH	19 (45)
Dyslipidemia	16 (38)
COPD	3 (7)
CD	1 (2)
ECKD	4 (10)
Deaths	3 (7)
	Laboratory at the admission
	Mean ± SE
Age	62 ± 13
BMI (kg/m^2^)	29 ± 4
Temperature (°C)	36.6 ± 0.46
PaO_2_	79.4 ± 38.4
PCO_2_	33.1 ± 7.8
PaO_2_/FiO_2_	123 ± 58.2
SpO_2_/FiO_2_	123.6 ± 52
HR	77 ± 20
MAP (mmHg)	79 ± 11
Glucose (70–105 mg/dL)	160.1 ± 77
Urea	56.8 ± 53
Ureic nitrogen	25.5 ± 22.8
Total cholesterol (mg/dL)	138.3 ± 38.4
Triglycerides (mg/dL)	155.2 ± 81.7
HDL (mg/dL)	33.1 ± 8.3
LDL (mg/dL)	75.7 ± 30.1
LDH (UI/L)	314.7 ± 100.2
TB	0.66 ± 0.20
BD	0.22 ± 0.09
Leukocytes (3.56–10.3 × 10^3^/μL)	11.4 ± 4.8
Lymphocytes (0.99–3.24 × 10^3^/μL)	0.67 ± 0.40
Platelets (1,50,000–5,00,000 × 10^3^/μL)	243.07 ± 87
Ferritin (11–307 ng/mL)	836.80 ± 757
	Laboratory at the admission
	Median (min–max)
CRP (1–3 mg/L)	151 (32–384)
Index N/L	13 (1–106)
IL-6 (pg/mL)	117.04 (7.8–638.5)
D-Dimer (0–0.24 μg/mL)	900 (210–34920)

*PaO_2_ = arterial blood oxygen pressure, BMI = body mass index, HR = heart rate, MAP = mean arterial pressure, HDL = high-density lipoproteins, LDL = low-density lipoproteins, LDH = lactic dehydrogenase, TB = total bilirubin, DB = direct bilirubin, IL = interleukin, N/L = neutrophil/lymphocyte, DM = diabetes mellitus, SAH = systemic arterial hypertension, CD = cardiovascular disease, COPD = chronic obstructive pulmonary disease, ECKD = end-stage chronic kidney disease, CRP = C-reactive protein, FiO_2_ = inspired fraction of oxygen, PCO_2_ = partial pressure of carbon dioxide, SpO_2_ = oxygen saturation.*

Three patients died (1.2%), out of which one developed an infection by pseudomonas. Of the other two patients who died without developing septic shock, one had end-stage chronic kidney failure and the other had an acute myocardial infarction. There were no patients with cancer, liver disease or previous autoimmune diseases or infection associated with bacteria and fungi. The demographic characteristics of the patients are shown in Table 1.

The 42 patients required invasive mechanical ventilation at the time of admission. The days required of invasive mechanical ventilation and the length of stay in the ICU had a median of 13 days with a minimum of 2 and a maximum of 30 days.

A total of 22 control patients were examined, of which 11 (50%) were men and 11 (50%) were women. HS had an average age of 51 ± 10 years. The values the CT, TG, HDL, and LDL are shown in Table 2. In these subjects, there was no suspicion of inflammatory disease or presence of degenerative disorders such as thyroid and autoimmune diseases. Diabetes mellitus, dyslipidemia, and arterial hypertension were not present.

**TABLE 2:** Age range and serum biochemical of the healthy subjects.

**Variable**	**Median (Min–Max)**
Age	54 (31–64)
CT (mg/dL)	168.5 (107–190)
TG (mg/dL)	152.5 (130.6–236.6)
HDL (mg/dL)	37.3 (29.4–48.5)
LDL (mg/dL)	89.3 (37–123.3)

*The healthy subjects were 11 women and 11 men. The values are expressed in median with Min-Max range. CT = cholesterol, TG = triglycerides, HDL = high density lipoprotein, LDL = low density lipoprotein.*

### Total Fatty Acids, FA of the PL, and NEFAS

The independent TFA percentage of each FA in the plasma of HS and the COVID-19 patients showed that the palmitic (PA), palmitoleic, stearic, γ-linoleic (γ-LA) dihomo-γ-linolenic (D-γ-LA) and eicosapentaenoic (EPA) fatty acids were decreased in the patients (*p* ≤ 0.009); however, oleic (OA), linoleic (LA), and arachidonic acid (AA) were increased (*p* = 0.001, 0.03, and 0.02, respectively) in COVID-19 patients (Table 3). When this FAs were analyzed by the unsaturation grade, the results show a decreased in PA and stearic acid proportions resulted in a statistically significant decrease in the total saturated fatty acids (SFAs) (*p* = 0.001). However, the increase in OA, despite the decrease in the palmitoleic acid, resulted in a rise in the total monounsaturated fatty acids (MUFAs) (*p* = 0.001). The polyunsaturated fatty acids (PUFAs) n-3 were reduced due to the decrease observed in the EPA and docosahexaenoic acid (DHA) (*p* = 0.001). However, PUFAs (n-6) were increased due to the high percentage in LA and AA (*p* = 0.02) in the COVID-19 patients (Table 4). These results suggest an alteration in the profile of the FA in COVID-19 patients. In the desaturation process of the FA, the desaturases are especially important, therefore some modification in this process may modify the percentage of the FA profile. The indirect determination of the desaturation process consists in dividing the products by the substrates. Table 5 shows that the ratios of palmitoleic/palmitic acids (C16:1n-7/C16:0) and γ-linoleic/linoleic acids (LAs) (γ-C18:3n-6/C18:2n-6), docosahexaenoic/eicosapentaenoic acids (C22:6n-3/C20:5n-3), that are usually used as indexes of the Δ^9^- and Δ^6^-desaturation activities for PA and D-γ-LA, showed a significant decrease in the COVID-19 patients (*p* = 0.001 and 0.005, respectively). In addition, the ratios of OA/stearic acids (C18:1n-9/C18:0) and arachidonic/dihomo-γ-linoleic (C20:4n-6/C20:3n-6), that are usually used as indexes of the Δ^9^- and Δ^5^-desaturation activities for stearic acid and AA were increased (*p* = 0.001), in COVID-19 patients in comparison to the HS.

**TABLE 3:** Total fatty acid compositions in the plasma from the healthy subjects and COVID-19 patients.

**Fatty acid**	**Healthy subjects**	**COVID-19 patients**	***p***
C16:0	35.78 ± 0.60	**30.57 ± 0.64**	**0.001**
C16:1	5.07 ± 0.28	**3.16 ± 0.20**	**0.001**
C18:0	13.50 ± 0.38	**7.84 ± 0.21**	**0.001**
C18:1n-9	18.80 ± 0.73	**28.82 ± 0.61**	**0.001**
C18:2n-6	22.01 ± 0.51	**24.26 ± 0.69**	**0.03**
γ-C18:3n-6	0.23 ± 0.02	**0.21 ± 0.03**	**0.009**
α-C18:3n-6	0.35 ± 0.02	0.43 ± 0.03	NS
C20:3n-6	0.60 ± 0.03	**0.40 ± 0.08**	**0.001**
C20:4n-6	2.47 ± 0.16	**3.17 ± 0.24**	**0.02**
C20:5n-3	0.70 ± 0.08	**0.20 ± 0.04**	**0.001**
C22:6n-3	0.20 ± 0.06	0.11 ± 0.03	NS

*Data represent the weights of each individual FA/weights of total FAs as percentages (mean ± SE) Fatty acid nomenclature: C16:0, palmitic acid; C16:1n-7, palmitoleic acid; C18:0, stearic acid; C18:1n-9, oleic acid; C18:2n-6, linoleic acid; γ-C18:3n-6, γ-linolenic acid; α-C18:3n-3, α-linolenic acid; C20:3n-6, dihomo-γ-linolenic acid; C20:4n-6, arachidonic acid; C20:5n-3 eicosapentaenoic acid; C22:6n-3, docosahexaenoic acid. The bold values indicate significant changes.*

**TABLE 4:** Total fatty acid composition of saturated, monounsaturated, and polyunsaturated in the plasma from the healthy subjects and COVID-19 patients.

**Fatty acid**	**Healthy subjects**	**COVID-19 patients**	***p***
SFA	49.10 ± 0.83	**38.65 ± 0.70**	**0.001**
MUFA	23.82 ± 0.70	**32.09 ± 0.61**	**0.001**
PUFA (n-3)	0.91 ± 0.11	**0.31 ± 0.05**	**0.001**
PUFA (n-6)	25.94 ± 0.53	**28.19 ± 0.82**	**0.02**

*SFA = saturated fatty acid, MUFA = monounsaturated fatty acids, PUFA = polyunsaturated fatty acids. Data are mean ± SE (healthy subjects *n* = 22) (COVID-19 patients *n* = 42). The bold values indicate significant changes.*

**TABLE 5:** Indirect desaturation indexes of the desaturases in the total fatty acids from healthy subjects and COVID-19 patients.

**Desaturation index**	**Healthy subjects**	**COVID-19 patients**	***p***
C16:1n-7/C16:0 (Δ^9^)	0.14 ± 0.007	**0.10 ± 0.006**	**0.001**
C18:1n-9/C18:0 (Δ^9^)	1.43 ± 0.08	**3.82 ± 0.13**	**0.001**
γ-C18:3n-6/C18:2n-6 (Δ^6^)	0.010 ± 0.001	**0.009 ± 0.001**	**0.005**
C20:4n-6/C20:3n-6 (Δ^5^)	4.88 ± 0.62	**12.81 ± 2.19**	**0.001**
C22:6n-3/C20:5n-3 (Δ^5^)	0.26 ± 0.06	0.33 ± 0.05	NS

*Fatty acid nomenclature: C16:0, palmitic acid; C16:1n-7, palmitoleic acid; C18:0, stearic acid; C18:1n-9, oleic acid; C18:2n-6, linoleic acid; γ-C18:3n-6, γ-linolenic acid; α-C18:3n-3, α-linolenic acid; C20:3n-6, dihomo-γ-linolenic acid; C20:4n-6, arachidonic acid; C20:5n-3 eicosapentaenoic acid; C22:6n-3, docosahexaenoic acid. Data are mean ± SE (healthy subjects *n* = 22) (COVID-19 patients *n* = 42). The bold values indicate significant changes.*

**FIGURE 1:**
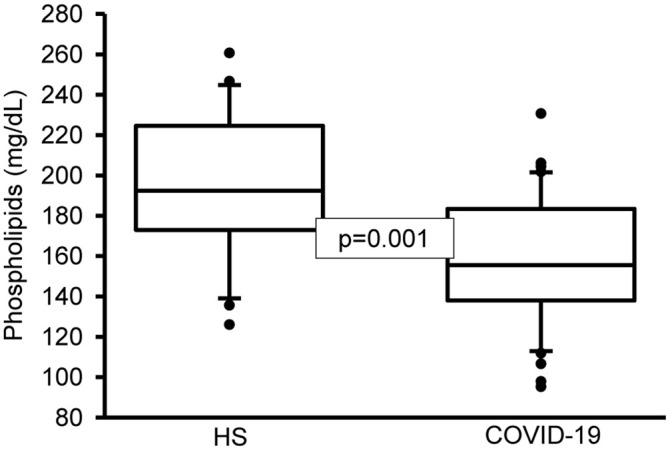
Total phospholipids in plasma of HS and COVID-19 patients. The main phospholipids are phosphatidylserine, phosphatidylcholine, phosphatidylethanolamine, sphingomyelin, and phosphatidylinositol. A decrease significant is observed in COVID-19 patients vs. HS. Statistical significance was determined by the Mann–Whitney rank sum test followed by the normality test (Shapiro-Wilk). Values expressed represent the median and Min–Max range (*n* = 42 COVID-19 patients and 22 HS). COVID-19 = coronavirus disease-19, HS = healthy subjects.

On another hand, the analysis by colorimetry of the TPL showed a decrease in the COVID-19 patients (*p* = 0.001, Figure 1). However, the analysis of the FA that make up the PL by gas chromatography showed that the PA, stearic, and D-γ-LA were increased (*p* = 0.001). Nevertheless, the OA, LA, and γ-LA percentage were decreased (*p* = 0.01 and 0.001, respectively) in the COVID-19 patients (Table 6). Therefore, the increased PA and stearic acid proportions resulted in a statistically significant increase in the SFAs of the FAPL (*p* = 0.001). However, the decrease in OA contributed to elevate the total MUFAs n-3 (*p* = 0.009). Moreover, the polyunsaturated FAs PUFAs n-6 were reduced due to the decrease observed in the LA and γ-LA (*p* = 0.001), in the COVID-19 patients (Table 7). In addition, the analysis of the indexes of the Δ^9^- and Δ^5^-desaturation activities associated with the ratios of C16:1n-7/C16:0, C18:1n-9/C18:0 and C20:4n-6/C20:3n-6 respectively, showed a significant decrease (*p* = 0.006 and 0.001, respectively, Table 8).

**TABLE 6:** Fatty acid compositions of the phospholipids in plasma from the healthy subjects and COVID-19 patients.

**Fatty Acid (%)**	**Healthy subjects**	**COVID-19 patients**	***p***
C16:0	26.34 ± 0.62	**33.47 ± 0.71**	**0.001**
C16:1	3.20 ± 0.31	2.78 ± 0.26	NS
C18:0	5.57 ± 0.21	**9.77 ± 0.32**	**0.001**
C18:1n-9	26.81 ± 0.84	**24.18 ± 0.57**	**0.01**
C18:2n-6	33.18 ± 0.89	**23.96 ± 0.62**	**0.001**
γ-C18:3n-6	0.31 ± 0.04	**0.21 ± 0.03**	**0.001**
α-C18:3n-6	0.48 ± 0.03	0.52 ± 0.07	NS
C20:3n-6	1.40 ± 0.16	**2.61 ± 0.17**	**0.001**
C20:4n-6	2.61 ± 0.19	2.35 ± 0.13	NS
C20:5n-3	0.03 ± 0.01	0.06 ± 0.02	NS
C22:6n-3	0.01 ± 0.009	0.04 ± 0.01	NS

*Data represent the weights of each individual FA/weights of total FAs as percentages (mean ± SE) Fatty acid nomenclature: C16:0, palmitic acid; C16:1n-7, palmitoleic acid; C18:0, stearic acid; C18:1n-9, oleic acid; C18:2n-6, linoleic acid; γ-C18:3n-6, γ-linolenic acid; α-C18:3n-3, α-linolenic acid; C20:3n-6, dihomo-γ-linolenic acid; C20:4n-6, arachidonic acid; C20:5n-3 eicosapentaenoic acid; C22:6n-3, docosahexaenoic acid. The bold values indicate significant changes.*

**TABLE 7:** Fatty acid compositions of the phospholipids; saturated, monounsaturated, and polyunsaturated in the plasma from the healthy subjects and COVID-19 patients.

**Fatty acid (%)**	**Healthy subjects**	**COVID-19 patients**	***p***
SFA	31.92 ± 0.58	**43.24 ± 0.82**	**0.001**
MUFA	30.01 ± 0.91	**26.96 ± 0.64**	**0.009**
PUFA (n-3)	0.04 ± 0.01	0.10 ± 0.02	NS
PUFA (n-6)	38.00 ± 1.04	**29.68 ± 0.70**	**0.001**

*SFA = saturated fatty acid, MUFA = monounsaturated fatty acids, PUFA = polyunsaturated fatty acids. Data are mean ± SE (healthy subjects *n* = 22) (COVID-19 patients *n* = 42). The bold values indicate significant changes.*

**TABLE 8:** Indirect desaturation indexes of the desaturases in the fatty acids of the phospholipids from healthy subjects and COVID-19 patients.

**Desaturation index**	**Healthy subjects**	**COVID-19 patients**	***p***
C16:1n-7/C16:0 (Δ^9^)	0.11 ± 0.01	**0.08 ± 0.007**	**0.006**
C18:1n-9/C18:0 (Δ^9^)	4.91 ± 0.27	**2.61 ± 0.11**	**0.001**
γ-C18:3n-6/C18:2n-6 (Δ^6^)	0.009 ± 0.001	0.01 ± 0.002	NS
C20:4n-6/C20:3n-6 (Δ^5^)	2.69 ± 0.61	**1.20 ± 0.13**	**0.001**
C22:6n-3/C20:5n-3 (Δ^5^)	0.19 ± 0.19	0.39 ± 0.13	NS

*Fatty acid nomenclature: C16:0, palmitic acid; C16:1n-7, palmitoleic acid; C18:0, stearic acid; C18:1n-9, oleic acid; C18:2n-6, linoleic acid; γ-C18:3n-6, γ-linolenic acid; α-C18:3n-3, α-linolenic acid; C20:3n-6, dihomo-γ-linolenic acid; C20:4n-6, arachidonic acid; C20:5n-3 eicosapentaenoic acid; C22:6n-3, docosahexaenoic acid. Data are mean ± SE (healthy subjects *n* = 22) (COVID-19 patients *n* = 42). The bold values indicate significant changes.*

**FIGURE 2:**
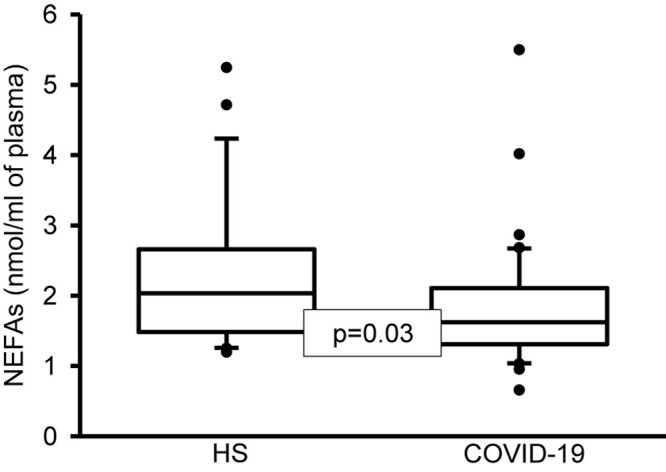
Total NEFAs in plasma of healthy subjects and COVID-19 patients. A decrease significant is observed in COVID-19 patients vs. HS. Statistical significance was determined by the Mann–Whitney rank sum test followed by the normality test (Shapiro-Wilk). Values expressed represent the median and Min–Max range (*n* = 42 COVID-19 patients and 22 HS). COVID-19 = coronavirus disease-19, HS = healthy subjects, NEFAS = non-esterified fatty acids.

In addition, the analysis by colorimetry of the NEFAs showed a decrease in the COVID-19 patients (*p* = 0.03, Figure 2). However, the analysis of the FA that make up the TNEFAs by gas chromatography showed that the palmitoleic, stearic, D-γ-LA, EPA and DHA were decreased (*p* = 0.04, 0.005, 0.003, 0.001, and 0.04, respectively). However, the OA and LA percentage were increased (*p* = 0.002 and 0.02, respectively) (Table 9). Moreover, the decrease in the percentage of the stearic acid resulted in a tendency to reduce the SFAs of the TNEFAs (*p* = 0.07). However, the decrease in EPA and DHA contributed to decrease the total MUFAs n-3 (*p* = 0.001) in the COVID-19 patients (Table 10).

**TABLE 9:** Fatty acid compositions of the NEFAs in the plasma from the healthy subjects and COVID-19 patients.

**Fatty acid (%)**	**Healthy subjects**	**COVID-19 patients**	***p***
C16:0	34.20 ± 0.85	33.60 ± 0.86	NS
C16:1	4.63 ± 0.94	**2.58 ± 0.29**	**0.04**
C18:0	14.55 ± 0.47	**12.07 ± 0.57**	**0.005**
C18:1n-9	21.11 ± 1.20	**25.20 ± 0.94**	**0.002**
C18:2n-6	15.61 ± 0.52	**17.92 ± 0.82**	**0.02**
γ-C18:3n-6	0.87 ± 0.16	0.73 ± 0.12	NS
α-C18:3n-6	0.93 ± 0.22	0.79 ± 0.15	NS
C20:3n-6	6.01 ± 0.75	**3.82 ± 0.36**	**0.003**
C20:4n-6	1.54 ± 0.11	1.43 ± 0.17	NS
C20:5n-3	0.41 ± 0.11	**0.07 ± 0.02**	**0.001**
C22:6n-3	0.09 ± 0.046	**0.02 ± 0.01**	**0.04**

*Data represent the weights of each individual FA/weights of total FAs as percentages (mean ± SE) Fatty acid nomenclature: C16:0, palmitic acid; C16:1n-7, palmitoleic acid; C18:0, stearic acid; C18:1n-9, oleic acid; C18:2n-6, linoleic acid; γ-C18:3n-6, γ-linolenic acid; α-C18:3n-3, α-linolenic acid; C20:3n-6, dihomo-γ-linolenic acid; C20:4n-6, arachidonic acid; C20:5n-3 eicosapentaenoic acid; C22:6n-3, docosahexaenoic acid. The bold values indicate significant changes.*

**TABLE 10:** Fatty acid compositions of the NEFAs; saturated, monounsaturated, and polyunsaturated in the plasma from the healthy subjects and COVID-19 patients.

**Fatty acid (%)**	**Healthy subjects**	**COVID-19 patients**	***p***
SFA	48.75 ± 1.05	**45.68 ± 1.06**	**0.07**
MUFA	25.74 ± 1.14	27.79 ± 1.07	NS
PUFA (n-3)	0.51 ± 0.12	**0.09 ± 0.03**	**0.001**
PUFA (n-6)	24.98 ± 0.73	24.71 ± 0.85	NS

*SFA = saturated fatty acid, MUFA = monounsaturated fatty acids, PUFA = polyunsaturated fatty acids. Data are mean ± SE (healthy subjects *n* = 22) (COVID-19 patients *n* = 42). The bold values indicate significant changes.*

## Discussion

Lipid molecules such as cholesterol (CT) and PL make up to 50% of the mass of most cell membranes. PL have a polar head and two FAs of different lengths ranging from 14 to 24 carbon atoms. Normally one of these FA is unsaturated and the other one is saturated, allowing the PL molecule to pack with other PL determining the fluidity of the membrane (Heaton and Randall, 2011). FA are the main components of the PL, and their metabolisms is very import for the survival of the cell. FA metabolism is as important for viruses as it is for eukaryotic cells. However, since the virus does not have the enzymatic pathways for the synthesis of FA, they sequester the machinery from the host cells, and manipulate the host’s cell lipid metabolism to facilitate their assembly and replication without considering the consequences for the host. One of the strategies of the virus is to reprogram the host’s FA metabolism to provide for the required FA molecules for the synthesis of the virion replication membranes (Heaton and Randall, 2011). The isomers of the transcription factor sterol regulatory element binding protein (SREBP) are involved in this metabolic reprogramming. As an example of this reprogramming, the MERS-CoV and flavivirus may manipulate host cellular lipid metabolism and reprogram the *de novo* SREBP dependent lipogenesis pathway, to ensure its replication (Pombo and Sanyal, 2018). The analysis of the plasma lipids from patients with the Ebola virus disease show that lipids are essential for viral structural components of the membrane, signaling molecules, and as energy sources. This suggests that the alteration in the lipid homeostasis of host cells is a viral strategy for creating a proper environment for replication (Murakami et al., 2017). Therefore, the aim of this work was to evaluate changes in the FA profile and to evaluate the participation of the desaturases in plasma of patients with severe pneumonia by SARS-CoV-2.

Palmitic acid and stearic acid are the saturated FA that are most abundant in the PL of the cell membrane. Our results show that these FA were significantly decreased in plasma from COVID-19 patients. However, the total PL were increased. These alterations may result from the requirement of lipid chains by the SARS-CoV-2 to conserve cysteine residues that are located adjacent to the transmembrane sections of the virus spike and envelope proteins (Tanner and Alfieri, 2021). Lipid addition occurs through the process of cysteine palmitoylation. Cysteine palmitoylation adds PA, stearic acid or AA moieties to cysteine residues that dynamically increase the affinity for cellular membranes and hydrophobic pockets on neighboring proteins or protein domains. Besides, protein palmitoylation promotes protein shuttling between different membrane compartments (Tanner and Alfieri, 2021). Furthermore, host membranes need to be repaired through the synthesis of FA since in the viral budding process, the virion produces perforations in the cell membranes. FA originate in the cytosolic monolayer of the membrane of the endoplasmic reticulum (ER), and they are subsequently translocated by the binding proteins of the FA by the process called flip-flop (Storch and Corsico, 2008). In addition, the decrease in palmitoleic acid could be the result of the decrease in the activity of the Δ^9^-desaturase, since it does not have enough substrate such as the PA. This was observed in our results. The PA, palmitoleic acid, OA percentage, and the activity of the Δ^9^-desaturase is also increased in obese subjects, metabolic syndrome models and in Marfan syndrome patients (Soto et al., 2016).

The desaturation process is the result of the activity of desaturases which are key enzymes in the biosynthesis of the MUFA, from of the saturated FA. They introduce a *cis* double bond into the FA hydrocarbon chains (Wang et al., 2020). These enzymes contribute to the control of FA-dependent structural disorders of the membrane (Verlengia et al., 2003), and these disorders may interfere with accessibility to receptors and may modify membrane ionic transport and cellular enzymatic activities (Das, 2010).

Oleic acid is an 18-carbon lipid with one double bond in the *cis* position, which is found in a high concentration in plasma of obese, hypertensive, and diabetic subjects and in patients with Marfan syndrome (Egan et al., 1999; Soto et al., 2016). It was also increased in our series of patients. OA modulates cytokine release, apoptosis, necrosis, oxidative stress (OS), and it stimulates iNOS while lowering phosphorylated eNOS expression in *in vitro* and *in vivo* studies (Cury-Boaventura et al., 2006). The proportion of OA is the result of the Δ^9^-desaturase activity upon stearic acid, and the activity of this enzyme can be calculated indirectly by the rate of conversion resulting from the addition of the products divided by the substrates (Høstmark and Haug, 2013). Our results shown that the Δ^9^-desaturases activity on the PL was decreased, but the TFA was increased in the plasma of patients with COVID-19. The desaturation process of FA by this enzyme is an important factor in the regulation of the FA of the membrane and may play a role in many disorders (Priante et al., 2005). Therefore, our result suggests that the desaturation process of the Δ^9^-desaturase is altered by the SARS-CoV-2 infection, resulting in an increase of OA. The elevation of the levels of OA is fundamental in the evolution of the ALI and ARDS by SARS-CoV-2 infection, since it participates in the inflammatory process, by over expressing iNOS and cyclooxygenase 2 (COX2) and generating nitrosative stress and the cytokine storm (Fang et al., 2009). Furthermore, OA induces the expression of COX 2, since an injection of OA increases the prostanoids derived from COX 2 in the pulmonary artery. This was associated with the presence of edema in patients with sepsis (Selig et al., 1987). Several studies have also shown that OA and AA decrease the levels of reduced glutathione and modulate the expression of genes related to OS including superoxide dismutase, glutathione reductase and glutathione-S-transferase (Verlengia et al., 2003).

The conversion from LA can occur after desaturation and elongation in the ER (Storch and Corsico, 2008). In this process, LA is converted to γ-linoleic acid (γ-LA) by the Δ^6^-desaturase and elongated to dihomo-γ-linoleic acid (D-γ-LA) (Hagve et al., 2001). This intermediary form is then desaturated by Δ^5^-desaturase and elongated to AA (Das, 2010). Our results shown that the Δ^6^-desaturase activity on the TFA decreased but there was not a significant change in PL in the plasma of the COVID-19 patients. This results in an increase in the LA and a decrease in TFA and PL.

In addition, SARS-CoV-2, damages mitochondrial function with the subsequent release the cytochrome C (Soria-Castro et al., 2020). Cytochrome C is part of the complex IV of the electron transport chain in the inner mitochondrial membrane. This membrane is rich in cardiolipin, a phospholipid formed by four molecules of LA. The damage to the mitochondria could in part explain the observed increase in this acid in the TFA percentage in the plasma of COVID-19 patients. This could also favor the decrease the Δ^6^-desaturase activity, which results in the decrease of γ-LA. In addition, reduction of LA is frequently found in hypertensive patients and hypertensive animal models, but when LA is administered, there is a decrease the blood pressure (Lee et al., 2012). These findings suggest that the increase in the percentage of this FA could participate in the decrease of blood pressure in the ALI or ARDS associate to infection by SARS-CoV-2.

Another FA that was found to be increased in COVID-19 patients was D-γ-LA, an intermediate in the AA biosynthesis (Das, 2010). The increase in the PL and the decrease of the TFA in the plasma reflect an alteration in the Δ^5^-desaturase activity in the COVID-19 patients. High glucose alters the activity of Δ^5^-desaturase, that modifies the percentage D-γ-LA. The infection by SARS-CoV-2 leads to anaerobic glycolysis (Soria-Castro et al., 2020), and this is associated with high glucose levels in COVID-19 patients (Hagve et al., 2001).

Arachidonic acid is stored within the cell membrane and is then released via the hydrolysis of PL by the phospholipase family including phospholipase A_2_ (PLA_2_) (de Lima et al., 2006). Different factors stimulate PLA_2_ such as an increase in the Ca^2+^ concentration through voltage dependent Ca^2+^ channels, the activation of K^+^-ATP channels and stimulation of PKC (Linkous and Yazlovitskaya, 2010). PLA_2_, is required for the replication of several coronaviruses including the MERS-CoV (Müller et al., 2018). Moreover, in the HCoV−229E infection there are increased levels of the PLA_2_−dependent glycerophospholipids and FA including LA and AA (Shen et al., 2020). The increased activity of PLA_2_ is associated with a high production of eicosanoids and oxylipins that could help propagate the infection and lead to thrombo-inflammatory complications (Thomas et al., 2020b). The AA released by PLA_2_ is then enzymatically oxidized through three enzymes: lipoxygenase (LOX), cytochrome P450 and cyclooxygenases (COXs), that result in leukotrienes, lipoxins, hydroxy eicosatetraenoic acids, epoxyeicosatrienoic acids and prostanoids synthesis which include thromboxane (TX), prostaglandins (PGs), and prostacyclins (Jamieson et al., 2017).

Our results show that the AA percentage in the TFA and the Δ^5^-desaturase activity was increased in plasma from COVID-19 patients. Several studies have associated high concentration AA with increased inflammation through increased production and secretion of TNF-α and PGE_2_ (Cubero and Nieto, 2012), and through suppression of the immune system (Calder, 2007). AA is also recognized as a second messenger that affects cellular functions by modulating intracellular signal transduction (Soto et al., 2018). In cultures of human cells, AA induces a significant increase in iNOS gene expression (Priante et al., 2005). The possible increase in the AA percentage could favor increase expression and/or activity of enzymes that depend on it such as COXs, LOX, cytochrome P450, and TX synthase. These enzymes are associated with the increase of their metabolites and participate in inflammation, thrombosis, and the cytokine storm. In addition, the AA percentage requires an appropriate threshold since the loss of its homeostasis causes adverse effects on the enzymatic pathways that depend on it, such as the PGE_2_ biosynthesis by COX2 (Robb et al., 2020). The biosynthesis in these pathways is low in non-inflamed tissues but increases immediately in acute inflammation prior to the infiltration by immune cells (Ricciotti and FitzGerald, 2011).

In contrast to our results, other studies by metabolomic and lipidomic assays have found that serum levels of AA in the PL were significantly reduced in COVID−19 patients and this was negatively associated with the severity of the disease (Robb et al., 2020), These studies have hypothesized that a deficiency in AA may enhance the susceptibility of an individual to be infected by SARS-CoV-2 and that the administration of this FA may improve the recovery process. Therefore, high levels of AA may be a defense mechanism of the host against the SARS-CoV-2 but at the same time it may cause inflammation and modulate the development of the cytokine storm (Das, 2020a). Furthermore, AA, and other unsaturated fatty acids like LA inactivate *in vitro* the process of envelopment of viruses including that of HCV, HBV, influenza, and other coronavirus species such as SARS, and MERS (Yan et al., 2019). According to Yan et al. the coronaviruses modulate and rearrange the host lipid profile to reach an intricate homeostasis optimized for its replication, and any exogenous manipulation that disrupts the equilibrium may interfere with the optimal replication of the viruses. Supplementation with LA and AA might disturb the LA–AA metabolism axis resulting in feedback reversion of lysophospholipids into PL through the Land’s cycle, and could limit viral replication (Yan et al., 2019). Therefore, this suggest that high levels of AA may be a defense mechanism of the host against the SARS-CoV-2, but at the same time, it may cause inflammation and modulate the production of the cytokine storm (Das, 2020b).

Regarding EPA and DHA which are polyunsaturated FA (PUFA with 3 or 6 double links), they are the main precursors for the synthesis of specialized pro-resolving mediators (SPMs) known as resolvins, maresins, protectins, and lipoxins. These mediators inhibit the synthesis of the pro-inflammatory cytokines that participate in the cytokine storm through the COX enzymes and LOX which down regulate the NF-kB pathway (Basil and Levy, 2016). Our results show that EPA were significantly decreased and that DHA tended to decrease in COVID-19 patients in the TFA. However, there was a significant decrease in the NEFAs. This suggests that in the acute inflammation associated to SARS-CoV-2, these FA are decreased. In the light of our results, the administration of EPA and DHA could be very import in the prevention and management of COVID-19. In a case of acute inflammation by activation of NF-kB, SPMs activate PPAR-γ and inhibit NF-kB. Consequently, the production of pro-inflammatory cytokines is reduced and the cytokine storm present in COVID-19 may be prevent (Weill et al., 2020). In addition, numerous studies have shown that EPA and DHA modulate inflammation. For example, EPA is considered as a more potent inhibitor than DHA for the inflammatory response in human asthmatic alveolar macrophages and is efficient in reducing pro inflammatory eicosanoids derived from the AA pathway (Kendall et al., 2019).

Although in this discussion we addressed the role of each FA independently, they can be grouped by the number of double links in their hydrocarbon chain into saturated (SFA without links doubles), monounsaturated (MUFA with a links double) and PUFA n-3 and -6. The increase in the SFA, MUFA, and PUFA n-3 and -6 reflect changes in membrane composition which interferes with accessibility to receptors and modifies membrane ion transport and enzyme activity. PUFAs n-3 modulate membrane rafts where angiotensin-converting enzyme 2 (ACE2) and TMPRSS2 are expressed and decrease the interaction between the virus and the host cell (Wassall et al., 2018). In addition, PUFAs n-3, are transported in plasma or by lipoproteins, and they serve as precursors for the production of SPMs by macrophages and neutrophils. Treatment with PUFAs increases the SPMs levels in human asthmatic alveolar macrophages (Mickleborough et al., 2009). Our results show an alteration in the percentages of the SFA, MUFA and PUFA n-3 and -6 in COVID-19 patients. These changes could be attributed to the type of diet ingested or to the different comorbidities such as dyslipidemia. However, this is unlikely because all the patients had COVID-19, and therefore, the alterations in FA metabolism may be associated with the infection by the virus.

The alteration in the FA metabolism can also contribute to the hypolipidemia reported in COVID-19 (Wei et al., 2020). In the hypolipidemia associated to SARS, there is a lower level of TC (Song et al., 2004). Our data demonstrate that COVID-19 patients develop hypolipidemia which is represented by low TFA, TPL, TNEFAs, CT, HDL, and LDL levels. Hypolipidemia is a rare condition, and it can be caused by a genetic alteration or by secondary factors such as a viral infection (Baker et al., 2010). Our data coincide with other studies. For example, in patients infected with dengue virus there is also a decrease in serum LDL levels (Lima et al., 2019), and patients in the cirrhosis phase of hepatitis B have lower levels of HDL and LDL (Cao et al., 2019). SARS-COV-2 may damage liver function and thereby reduce HDL and LDL biosynthesis by diminishing the CT efflux and transport (Funderburg and Mehta, 2016), but the virus may also alter vascular permeability, causing a leakage of CT molecules into tissues, such as alveolar spaces, to form an exudate (Wei et al., 2020).

An alteration in CT levels is associated with lipid rafts, subdomains of the cellular membrane that can harbor ACE2 receptors for the S-protein of SARS-CoV-2 to anchor (Glende et al., 2008). Lipid rafts are subdomains of the plasma membrane enriched in CT and glycosphingolipids with large proportion of LA. The analysis by cryo-electron microscopy of the structure of spike S glycoprotein of SAR-CoV-2 reveals that the receptor binding domains are tightly bound to the free LA which is essential for the entry of the virus to the host cell (Toelzer et al., 2020). Furthermore, lipid rafts are important for the interaction between the S protein and ACE2 receptor and for facilitating the process of viral endocytosis (Baglivo et al., 2020). When the cell needs CT for membranes synthesis, it synthesizes and overexpresses the HDL and LDL receptors on the membrane. The binding of lipoproteins with their receptor leads to the internalization of CT by endocytosis. The decrease of CT, HDL, and LDL, in COVID-19 patients (Wei et al., 2016) could be associated to the formation of viral membrane and to reparation of the host’s membrane after of the budding process to avoid the loss of cell homeostasis. Since the membranes contain especially high amounts of CT, up to a proportion of more than one CT molecule for each PL molecule, this reinforces the permeable characteristic of the lipid bilayer. CT is also present in the viral envelope and is a key component for the entrance of the virus, since it is involved in binding and altering the oligomeric status of the N-terminal fusion peptide of SARS-CoV (Meher et al., 2019). In addition, inflammation alters hepatic apolipoprotein gene expression, decreases plasma levels of lecithin CT acyltransferase, and alters HDL function, composition, and antioxidant capacity (Han et al., 2016). Impaired HDL antioxidant activity results in lipid oxidation, generating oxidized LDL (oxLDL) and oxidized HDL (oxHDL) which are potent activators of the oxLDL scavenge receptor LOX−1. This receptor induces inflammation and aggravates tissue damage. During acute inflammation, free radicals are generating that may lead to lipid hydroperoxides derived from the LOX pathway (Chavarría et al., 2021). These hydroxyl acids are metabolites that derivate from LA and AA, and may be esterified into CT esters, triacylglycerol, and PL in oxLDL, oxHDL which may contribute to hypertriglyceridemia (Sorokin et al., 2020). In addition, increased levels of oxLDL and oxHDL constitute a major independent risk factor for atherosclerotic cardiovascular disease and renal damage (Chawla et al., 2020).

The total fatty acids of phospholipids (TFAPL) are the building blocks for PL. They are synthesized in the liver and are necessary for lipoproteins, for membrane formation and for cell vesicular transport. The main PL are phosphatidylcholine, sphingomyelin, phosphatidylserine, phosphatidylethanolamine, and phosphatidylinositol (Kumar et al., 2020). These PL regulate several processes including growth, cell migration, adhesion, apoptosis, and inflammatory responses and mediate signal transduction and immune activation processes (Yusuf and Lina, 2018). Our results show a decrease in TPL in the plasma of the COVID-19 patients. This suggests that these PL could be used in the formation of both viral membrane and for reparation of the host’s own membrane after of the budding process. PL are also highly susceptible to oxidation by free radicals which results in oxidized PL (oxPL) and favor lipid peroxidation (Chavarría et al., 2021). The oxPL are recognized as important bioactive lipid mediators playing an active role as modulators in signaling events in inflammation, immunity, infection, and biomarkers of atherosclerosis and other pathologies (Rogero et al., 2020). The oxPL production is increased in the lungs of virus−infected humans and animals and oxPL induce macrophage cytokine production and ALI in mice. A study in SARS-CoV-2 infection shows a decrease in the PL and FAPL such as AA which was significantly elevated in HCoV-229E-infected cells (Shen et al., 2020).

In addition, triglycerides (TG) are especially important, and the COVID-19 patients show hypertriglyceridemia (Kovacevic et al., 2021). The identification of high levels of TG suggests an increase in adipose tissue lipolysis in COVID-19 patients (Lee et al., 2018). In our series, the COVID-19 patients showed an increase in TG and glucose. Although the exact mechanism for this increase is unknown, it could be related to an elevation in the anaerobic glycolysis caused by SARS-CoV-2 (Soria-Castro et al., 2020). As an example, the HCV induces lipid accumulation in hepatocytes by inhibiting microsomal TG transfer protein activity (Chen et al., 2016). However, in patients with sepsis, hypolipidemia is usually found, and the possible mechanism behind this lipid alteration is a tissue lipoprotein lipase inhibition, an upregulated hepatic TG production, disruption of the synthesis-utilization balance and interaction with cytokines or endotoxins (Ilias et al., 2014). TG are strongly up regulated, and adipose tissue is important in adapting to critical illness; in fact, during starvation, the lipolysis of adipose tissue increases (Ilias et al., 2014). These alterations can lead to an imbalance in non-esterified FA (NEFAs). In this regard, several studies with other types of viruses such as dengue, swine fever virus, HCV, HIV and HBV have reported these alterations (Villamor et al., 2018).

Our results also show a decrease in NEFAs in the plasma of the COVID19 patients. In this regard, other researchers have reported a decrease (Frasca et al., 2020; Thomas et al., 2020b), and others an increase (Shen et al., 2020) these results could be contradictory. However, the explanation could be, the dependance on the state of the infection process. In the early stages of infection more energy is needed to combat the physiological changes such as fever, muscle ache and headache which are associated with a pro-inflammatory cytokine storm. Therefore, more fat is metabolized into NEFAs with an increase in the rate of lipolysis by the hormone-sensitive lipase and decrease in lipogenesis. However, when the infection progresses, the previous situation aggravates leading to sepsis or septic shock which may result in a decrease in energy. Therefore, rapid diffusion of NEFAs through the phospholipid bilayer should be considered as a factor that favors the viral replication and cell membrane reparation. Eventually, FA metabolism becomes fully depressed with a decrease in the circulation of the NEFAs (Renli et al., 2012).

On another hand, A limitation of the present study is the absence of a control group matched by the presence of comorbidities such as obesity, diabetes, and hypertension, which may affect the results and the conclusion. In these conditions’ hyperlipidemia is usually found which contrasts with the hypolipidemia found in COVID-19 patients. In this regard, three non-essential FA and one essential FA have been previously found to be independently associated with blood pressure. Stearic acid was associated with lower blood pressure levels, while PA and eicosatrienoic acid were associated with higher blood pressure levels. D-γ-LA acid has also been associated with higher blood pressure levels (Simon et al., 1996). Likewise, insulin-dependent diabetes, is characterized by increases in OA and α-LA. In non-insulin-dependent diabetes, there are elevations in the levels of OA and total MUFA in the triglyceride fraction (Garaulet et al., 2011). There is also an increase in SFA levels and decrease in PUFA levels in phosphatidylcholine (Seigneur et al., 1994). Although a strong link between plasma lipids or hyperlipidemia and COVID-19 comorbidities such as cardiovascular diseases, diabetes mellitus or obesity, has been suggested which may worsen the prognosis of COVID-19 (Zhang et al., 2020), a systematic review and meta-analysis aimed to evaluate whether dyslipidemia affects the mortality and severity of COVID-19, found that there is an association between dyslipidemia and a poor outcome in patients which varied with age, male gender, and hypertension, but not with diabetes and cardiovascular diseases (Atmosudigdo et al., 2021). Again, it is important to note that in COVID-19 patients there is hypolipidemia instead of hyperlipidemia.

**FIGURE 3:**
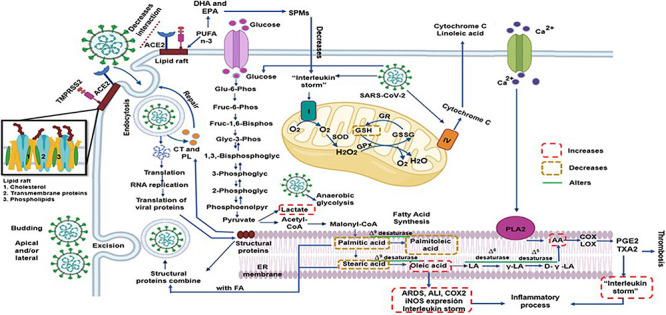
The SARS-CoV-2 can modulate and rearrange the host lipid profile to reach an intricate homeostasis which is optimal for viral replication and propagation. However, any exogenous manipulation that disrupts this the equilibrium might interfere with the replication of the virus such as high EPA and DHA concentration that decrease pro-inflammatory state being precursor of SPMs to and probably the AA however this FA might have two effects; increase inflammatory and thrombotic prostaglandins that would aggravate the infection by SARS-CoV-2 and/or decrease viral replication and propagation. AA = arachidonic acid, ACE2 = angiotensin converting enzyme 2, COX = cyclooxygenase, CT = cholesterol, D-γ-LA = dihomo-γ-linolenic acid, DHA = docosahexaenoic acid, EPA = eicosapentaenoic acid, FA = Fatty Acid, GPx = glutathione peroxidase, GR = glutathione reductase, GSH = glutathione, GSSG = oxidized glutathione, H2O2 = hydrogen peroxide, LA = linoleic acid, LOX = lipoxygenase, O_2_ = superoxide anion, PGE2 = prostaglandin E2, PL = phospholipids, PLA2 = phospholipase A_2_, PUFA = polyunsaturated fatty acids, SOD = superoxide dismutase, SPMs = specialized pro-resolving mediators, TXA2 = thromboxane A2, γ-LA = γ-linoleic acid.

In summary, the SARS-CoV-2 seems to modulate and rearrange the host lipid profile to reach an intricate homeostasis which is optimal for viral replication. Any exogenous manipulation that disrupts the equilibrium may interfere with this process. Figure 3 described this process.

## Conclusion

SARS-CoV-2 alters the FA metabolism. Changes are characterized by alterations in the desaturases that lead to a change in TFA, PL, and NEFAs profile. These alteration in the COVID-19 patients may favor the viral replication but at the same time, they are part of the defense system that the host cells metabolism provides in its eagerness to repair damage caused to cell membranes for the virus.

## Data Availability Statement

The datasets generated and analyzed during the current study are available from the corresponding author on reasonable request.

## Ethics Statement

The studies involving human participants were reviewed and approved by Ethical approval to perform the study was obtained from the local ethics committee on August 19, 2020 (Control-9867/2020, register REG. CONBIOETICA-09-CEI-011-20160627). A written informed consent for enrollment or consent to use patient data was obtained from each patient or their legal surrogate. The protocol was registered (TRIAL REGISTRATION: ClinicalTrials.gov Identifier: NCT 04570254). The patients/participants provided their written informed consent to participate in this study. Written informed consent was obtained from the individual(s) for the publication of any potentially identifiable images or data included in this article.

## Author Contributions

IP-T and VG-L designed the study and wrote the manuscript. VG-L revised and structured the manuscript. IP-T, MS, and ES-C made the laboratory determination. LM-P designed and made the graphical abstract. AP-C, RV-V, JD-C, HH-B, HC-S, LM-C, GA-E, FH, and OG-M treated and recruited the patients in the intensive care unit and collected the biochemical results. RM-V made the IL-6 determinations. MS designed the tables, figures, and performed and planned the statistical analysis. IP-T and MS contributed to the conceptualization and design of the project, methodology, and statistical analysis. All authors have read and agreed to the published version of the manuscript.

## Conflict of Interest

The authors declare that the research was conducted in the absence of any commercial or financial relationships that could be construed as a potential conflict of interest.
